# Combined Triglyceride–Glucose and Triglyceride–Glucose–Body Mass Index with B-Type Natriuretic Peptide for Enhanced Prediction of Major Adverse Cardiovascular Events in ST-Elevation Myocardial Infarction Patients: A Retrospective Cohort Study

**DOI:** 10.31083/RCM44062

**Published:** 2026-01-21

**Authors:** Jinyong Huang, Junyi Zhang, Linjie Li, Meiyan Chen, Yongle Li, Xiangdong Yu, Shaozhuang Dong, Qing Wang, Jun Chen, Qing Yang, Shaopeng Xu

**Affiliations:** ^1^Department of Cardiology, Tianjin Medical University General Hospital, 300052 Tianjin, China; ^2^Department of Cardiology, Tianjin Chest Hospital, 300222 Tianjin, China; ^3^Department of Emergency, Qilu Hospital of Shandong University, 250012 Jinan, Shandong, China

**Keywords:** ST-elevation myocardial infarction, triglyceride–glucose index, B-type natriuretic peptide, major adverse cardiovascular events, risk stratification, insulin resistance

## Abstract

**Background:**

Metabolic dysfunction significantly influences cardiovascular outcomes following ST-elevation myocardial infarction (STEMI). The triglyceride–glucose (TyG) index and triglyceride–glucose–body mass index (TyG–BMI) serve as surrogate markers of insulin resistance, whereas B-type natriuretic peptide (BNP) levels reflect cardiac dysfunction. However, the combined prognostic value of these biomarkers for predicting major adverse cardiovascular events (MACEs) in patients with STEMI remains underexplored.

**Methods:**

We conducted a retrospective cohort study of 1177 consecutive patients with STEMI who underwent percutaneous coronary intervention between August 2018 and December 2023. Patients were stratified into four groups based on the TyG index (cutoff: 7.2), TyG–BMI (cutoff: 186), and BNP level (cutoff: 300 pg/mL). The primary endpoint was MACEs, defined as a composite of all-cause mortality, nonfatal myocardial infarction, ischemia-driven repeat revascularization, heart failure hospitalization, and cerebrovascular events. Cox proportional hazards models with progressive adjustment were employed to assess independent and combined prognostic significance.

**Results:**

A total of 483 patients (41.0%) experienced MACEs during a median follow-up of 461 days (interquartile range (IQR): 79–672). Patients with both an elevated TyG index (≥7.2) and a high BNP concentration (≥300 pg/mL) demonstrated the highest cardiovascular risk profile and a more than twofold increased MACE risk (hazard ratio (HR) 2.18, 95% confidence interval (CI): 1.57–3.03; *p* < 0.001) compared with the reference group (those with a low TyG index and low BNP concentration). Similarly, patients with elevated TyG–BMIs (≥186) and BNP levels had an 81% increased risk (HR 1.81, 95% CI: 1.30–2.51; *p* < 0.001). Meanwhile, the combined TyG index + BNP model demonstrated superior predictive accuracy (area under the curve (AUC): 0.67) compared with the individual biomarkers and the established Global Registry of Acute Coronary Events (GRACE) score (AUC: 0.58). Subgroup analyses revealed particularly pronounced associations in older patients, females, and those with hypertension.

**Conclusions:**

The combination of the TyG index or TyG–BMI with BNP provides enhanced prognostic stratification for predicting MACEs in STEMI patients, offering superior discriminatory capacity compared with that of individual biomarkers. This integrated approach may facilitate personalized risk assessment and guide therapeutic decision-making in clinical practice.

## 1. Introduction

ST-elevation myocardial infarction (STEMI) represents the most severe form of acute coronary syndrome and is characterized by complete coronary artery occlusion and substantial myocardial necrosis. Despite significant advances in reperfusion strategies and evidence-based pharmacotherapy, STEMI patients continue to face a considerable risk of major adverse cardiovascular events (MACEs), with reported rates ranging from 10% to 20% annually following the index event [1, 2]. Accurate risk stratification remains paramount for optimizing therapeutic interventions and improving long-term cardiovascular outcomes.

Traditional risk assessment tools, including the Global Registry of Acute Coronary Events (GRACE) score and Thrombolysis in Myocardial Infarction (TIMI) risk score, primarily incorporate demographic, clinical, and procedural variables [3, 4]. However, these conventional models may not fully capture the complex pathophysiological processes underlying postinfarction cardiovascular risk, particularly the intricate interplay between metabolic dysfunction and cardiac stress responses.

Insulin resistance has emerged as a critical pathophysiological mechanism linking metabolic abnormalities to cardiovascular disease progression. Compared with the homeostatic model assessment of insulin resistance (HOMA-IR), the triglyceride–glucose (TyG) index serves as a reliable surrogate marker of insulin resistance with superior predictive capacity [5, 6]. Recent investigations have demonstrated significant associations between an elevated TyG index and adverse cardiovascular outcomes across diverse populations, including patients with acute coronary syndromes [7, 8]. Furthermore, the triglyceride–glucose–body mass index (TyG–BMI), which incorporates anthropometric parameters, may provide enhanced metabolic risk assessment by reflecting both insulin resistance and adiposity-related cardiovascular risk [9, 10].

However, the prognostic utility of single biomarkers remains limited. For instance, metabolic indicators such as the TyG index or TyG–BMI mainly reflect insulin resistance and obesity-related risk, whereas cardiac stress markers such as B-type natriuretic peptide (BNP) primarily capture the hemodynamic burden and ventricular dysfunction. Relying on a single dimension of risk information may fail to fully characterize the multifaceted pathophysiological processes after STEMI, thereby restricting predictive performance. In contrast, combined biomarker approaches integrate complementary mechanisms and provide a more comprehensive assessment, offering superior sensitivity and specificity in risk stratification and supporting more precise clinical decision-making.

In conjunction with metabolic risk assessment, BNP represents a well-established biomarker of cardiac dysfunction and hemodynamic stress. Elevated BNP levels reflect increased ventricular wall tension and volume overload and serve as powerful predictors of heart failure development and cardiovascular mortality following myocardial infarction [11, 12]. The prognostic utility of BNP has been consistently demonstrated across various cardiovascular conditions, with guideline recommendations supporting its clinical application for risk stratification and therapeutic monitoring [13].

The concept of integrated biomarker approaches for cardiovascular risk prediction has attracted considerable attention, as complex cardiovascular pathophysiology involves multiple interdependent mechanisms. The combination of metabolic markers with cardiac stress indicators may provide complementary prognostic information, potentially enhancing risk discrimination beyond individual biomarker assessment. However, the combined prognostic value of the TyG index, TyG–BMI, and BNP level for predicting MACEs in STEMI patients has not been comprehensively investigated.

Given the clinical importance of accurate risk stratification in STEMI management and the potential synergistic effects of metabolic and cardiac biomarkers, we hypothesized that the combination of the TyG index or TyG–BMI with BNP would provide superior prognostic discrimination for MACE prediction compared with individual biomarker assessment. Therefore, we conducted this comprehensive retrospective cohort study to (1) evaluate the individual prognostic significance of the TyG index, TyG–BMI, and BNP for MACE prediction in STEMI patients; (2) investigate the combined prognostic value of these biomarkers using systematic risk stratification approaches; (3) assess the incremental predictive capacity of integrated biomarker models compared to established risk scores; and (4) identify patient subgroups who may derive particular benefit from this combined biomarker approach.

## 2. Materials and Methods

### 2.1 Study Design and Patient Population

We conducted a retrospective cohort analysis involving STEMI patients who were admitted to Tianjin Medical University General Hospital between August 2018 and December 2023. The study protocol was approved by the Ethics Committee of Tianjin Medical University General Hospital (approval number: IRB2023-YX-301-01/2023) and adhered to the principles outlined in the Declaration of Helsinki. Owing to the retrospective nature of the study, the requirement for informed consent was waived.

The inclusion criteria were adults (≥18 years) diagnosed with definitive STEMI, as per the following standard criteria [14]: ischemic symptoms lasting ≥30 minutes, electrocardiographic evidence of ST-segment elevation (≥1 mm in at least two contiguous leads), or new left bundle branch block, and elevated cardiac troponin levels exceeding the 99th percentile. A total of 1480 consecutive patients were identified, with 303 exclusions based on predefined criteria such as lack of coronary angiography, severe organ dysfunction, or insufficient clinical data. The final cohort consisted of 1177 patients who underwent standardized evaluation and treatment protocols (**Supplementary Fig. 1**).

At the time of admission, baseline demographic and clinical characteristics were comprehensively recorded. Current smoking status was defined as the daily consumption of at least one cigarette within the 30 days prior to hospitalization [15]. The diagnosis of diabetes mellitus was established either through a prior confirmed diagnosis or through the use of glucose-lowering medications. Hypertension was identified according to one of the following criteria: (1) a documented clinical diagnosis, (2) the use of antihypertensive medications before admission, or (3) a new diagnosis made during the index hospitalization based on repeated blood pressure readings exceeding 140/90 mmHg.

### 2.2 Sample Size Estimation

Sample size calculations were performed on the basis of Cox proportional hazards models, assuming a clinically meaningful hazard ratio of 1.5 with 80% power at a two-sided significance level of 0.05. Previous studies have reported a cumulative MACE incidence of 15% over a two-year follow-up period [16, 17]. The final required sample size, after accounting for potential follow-up losses, was 708 patients, and our cohort of 1177 patients ensured adequate statistical power.

### 2.3 Data Collection and Laboratory Assessments

Data, including demographic information, medical history, and clinical parameters, were extracted from the patients’ electronic medical records. Laboratory analyses, performed at admission, included metabolic indices (e.g., fasting blood glucose and lipid profiles), renal function, and cardiac biomarkers (BNP and troponin). The TyG index was computed as TyG Index = ln(triglycerides (mg/dL) × fasting blood glucose (mg/dL))/2, and the TyG–BMI was derived by multiplying the TyG index by the body mass index (BMI).

### 2.4 Coronary Intervention and Follow-up

All patients underwent coronary angiography and subsequent percutaneous coronary intervention (PCI) per current guidelines [18]. The complexity of coronary lesions was assessed using the Synergy Between PCI With TAXUS and Cardiac Surgery (SYNTAX) scoring system by two experienced interventional cardiologists who were blinded to the patients’ clinical data. In cases of scoring discrepancies, a third cardiologist was involved to reach a consensus. The residual SYNTAX score (rSS) was then computed to quantify the untreated coronary disease burden after PCI. Both the initial SYNTAX score and the rSS have been shown to have prognostic value in previous studies [19]. Follow-up was conducted using electronic medical records and structured telephone interviews to ensure comprehensive event tracking. The MACE variable was defined as a composite of all-cause mortality, nonfatal myocardial infarction, ischemia-driven revascularization, hospitalization for heart failure, and cerebrovascular events.

### 2.5 Statistical Analysis

The primary outcome of interest was MACE occurrence, which was analyzed using multivariable Cox regression models with restricted cubic splines (RCSs) to assess nonlinear associations. RCSs were employed to allow for flexible modeling of continuous variables, capturing potential nonlinear relationships between the biomarkers and the outcome of interest. The knots for the RCS were placed at the 10th, 50th, and 90th percentiles of each continuous variable to ensure a balanced representation across the range of data. This method was specifically chosen to account for potential nonlinear trends, which are often observed in medical outcomes, and to provide more accurate and clinically relevant hazard ratios.

The study population was stratified on the basis of cutoff values for the TyG index (≥7.2), TyG–BMI (≥186), and BNP concentration (≥300 pg/mL), and Kaplan‒Meier curves were constructed to visualize survival differences across the stratified groups. The log-rank test was used to assess the statistical significance of differences in survival curves between groups, which provides a nonparametric method for comparing survival distributions.

The prognostic accuracy of these models was assessed using receiver operating characteristic (ROC) curve analysis to calculate the area under the curve (AUC), and the combined models were compared with individual biomarkers to establish risk scores. The AUC provides an aggregate measure of the model’s discriminative ability, and a comparison of the AUC values across the models was performed to determine whether the addition of biomarkers improved the prediction accuracy beyond traditional risk scores.

Model adjustments included demographic factors, clinical variables, coronary disease severity (SYNTAX score), and medical interventions, ensuring that potential confounders were accounted for in the analysis. To comprehensively assess the robustness of the associations, we constructed six progressively adjusted models as follows: Model 1 was the unadjusted model. Model 2 was adjusted for sex and age. Model 3 included the variables in Model 2, with the addition of heart rate, systolic blood pressure (SBP), diastolic blood pressure (DBP), current smoking status, hypertension, diabetes, stroke, left ventricular ejection fraction (LVEF), SYNTAX score, and rSS Model 4 was built upon Model 3 by further adjusting for the number of stents, antiplatelet therapy, statins, beta-blockers, angiotensin-converting enzyme inhibitor (ACEI)/angiotensin receptor blockers (ARBs)/angiotensin receptor and neprilysin inhibitor (ARNI), Proprotein convertase subtilisin/kexin type 9 (PCSK9) inhibitors, sodium-glucose cotransporter 2 (SGLT2) inhibitors, hemoglobin, platelet count, estimated glomerular filtration rate (eGFR), troponin T (TnT), and low-density lipoprotein cholesterol (LDL-C). Model 5 extended Model 4 by incorporating bootstrapping to enhance statistical robustness. Model 6 was performed using propensity score matching (PSM). A multivariable logistic regression model was applied to estimate the propensity score, adjusting for the covariates included in Model 4. Patients were matched 1:1 on the basis of their propensity scores using a greedy matching algorithm without replacement, with a caliper width set at 0.2 of the standard deviation of the log-transformed propensity score.

The proportional hazards assumption was verified using Schoenfeld residuals, and no significant violations were identified, indicating that the Cox regression model’s assumptions were met. Additionally, subgroup analyses were conducted to explore potential differences in risk prediction across subgroups on the basis of age, sex, diabetes status, and coronary complexity. These subgroup analyses help assess the heterogeneity of risk and evaluate the generalizability of the findings across different patient characteristics.

To further assess potential multicollinearity among the covariates in the multivariable models, collinearity diagnostics were performed using the variance inflation factor (VIF). A VIF value greater than 5 was considered indicative of significant multicollinearity.

All analyses were conducted using Stata version 16 (StataCorp, College Station, TX, USA). Two-sided *p*-values < 0.05 were considered to indicate statistical significance.

## 3. Results

### 3.1 Study Population and Baseline Characteristics

Between August 2018 and December 2023, our retrospective cohort study initially identified 1480 consecutive STEMI patients at Tianjin Medical University General Hospital. After excluding 76 patients due to inability to complete follow-up (all attributed to loss of contact, including invalid contact information or relocation), 1177 patients were enrolled for the final analysis. During a median follow-up period of 461 days (interquartile range: 79–672 days), 483 patients (41.0%) experienced MACEs. The distributions of individual MACE components, including all-cause mortality, nonfatal myocardial infarction, cerebrovascular events, heart failure hospitalization, and ischemia-induced revascularization, are summarized in **Supplementary Table 1**.

Baseline characteristics stratified by MACE occurrence are presented in **Supplementary Table 2**. Patients who developed MACEs were significantly older than those without MACEs (67 (59, 73) vs. 65 (54, 71) years, *p* < 0.001) and had a higher prevalence of cardiovascular comorbidities, including hypertension (73.3% vs. 64.0%, *p* < 0.001) and diabetes mellitus (37.5% vs. 24.1%, *p* < 0.001).

The MACE group exhibited greater coronary disease complexity, as evidenced by significantly higher SYNTAX scores [22.0 (17.0, 27.5) vs. 16.0 (11.0, 21.5), *p* < 0.001] and residual SYNTAX scores [10.0 (5.0, 15.0) vs. 5.0 (1.0, 8.0), *p* < 0.001], with higher proportions requiring multiple stent implantation (≥2 stents: 39.8% vs. 28.5%, *p* < 0.001). Laboratory analysis revealed that MACE patients had significantly elevated metabolic markers, including a higher TyG index [7.6 (7.2, 8.1) vs. 7.3 (7.0, 7.7), *p* < 0.001] and TyG–BMI index [191.5 (171.9, 219.7) vs. 183.2 (164.5, 206.9), *p* < 0.001], as well as impaired cardiac function reflected by elevated BNP levels [103.0 (28.1, 408.0) vs. 64.0 (22.0, 206.0) pg/mL, *p* < 0.001] and reduced eGFRs [92.2 (71.3, 111.6) vs. 99.5 (83.3, 116.8) mL/min/1.73 m^2^, *p* < 0.001]. These findings indicate that patients who experience MACEs present with more complex clinical profiles characterized by greater metabolic dysfunction, impaired cardiac function, and more extensive coronary artery disease.

All the covariates demonstrated acceptable collinearity (VIF <3), suggesting that there were no significant multicollinearity issues (**Supplementary Table 3**).

### 3.2 Individual Prognostic Value of the TyG Index, TyG–BMI, and BNP for MACEs

**Fig. 1.**
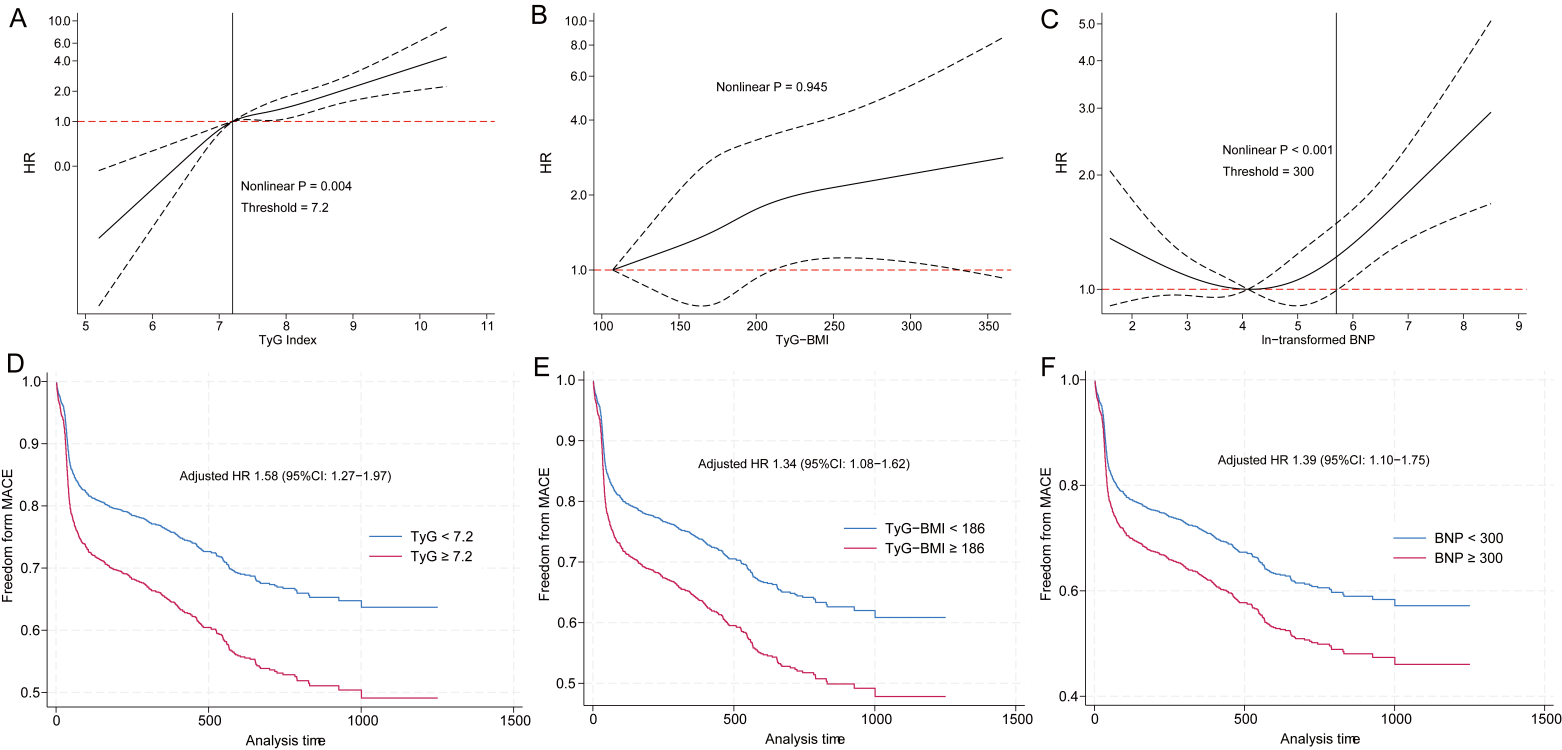
**Relationships between the TyG index, TyG–BMI, and BNP level and the risk of a MACE in patients with STEMI**. (A) Multivariable RCS analysis revealing the nonlinear relationship between the TyG index and MACE risk (inflection point: 7.2). (B) Multivariable RCS analysis demonstrating the predominantly linear association between TyG–BMI and MACE risk (optimal cutoff: 186). (C) Multivariable RCS analysis revealing the nonlinear relationship between BNP and MACE risk (inflection point: 300). (D) Kaplan‒Meier curves stratified by the TyG index (<7.2 vs. ≥7.2), showing significantly different MACE-free survival rates (HR 1.58, 95% CI: 1.27–1.97; *p* < 0.001). (E) Kaplan‒Meier curves stratified by TyG–BMI (<186 vs. ≥186), showing significantly divergent MACE-free survival rates (HR 1.34, 95% CI: 1.08–1.62; *p* < 0.001). (F) Kaplan‒Meier curves stratified by BNP level (<300 vs. ≥300), revealing substantial differences in MACE-free survival (HR 1.39, 95% CI: 1.10–1.75; *p* < 0.001). Shaded areas around the survival curves represent 95% confidence intervals. TyG, triglyceride–glucose; TyG–BMI, triglyceride–glucose–body mass index; BNP, B-type natriuretic peptide; MACE, major adverse cardiovascular event; STEMI, ST-segment elevation myocardial infarction; RCS, restricted cubic splines; HR, hazard ratio; CI, confidence interval.

The frequency distributions of the TyG index, TyG–BMI index, and BNP level are illustrated in **Supplementary Fig. 2**. Multivariable restricted cubic spline analysis revealed distinct relationships between these biomarkers and MACE risk (Fig. 1A–C). Both the TyG index and BNP level exhibited nonlinear relationships with MACE risk, with significant thresholds at a TyG index ≥7.2 and a BNP level ≥300 pg/mL, beyond which the hazard ratio increased substantially. In contrast, the TyG–BMI demonstrated a predominantly linear association with MACE risk, with an optimal cutoff value of 186.

Kaplan‒Meier survival curves with corresponding log-rank test results (Fig. 1D–F) illustrated differential MACE risk stratification by these biomarkers according to their respective cutoff points. Compared with those with lower values, those with an elevated TyG index (≥7.2) had a significantly greater risk of MACEs (HR 1.58, 95% CI: 1.27–1.97; *p* < 0.001). Similarly, subjects with an elevated TyG–BMI (≥186) had a significantly increased risk of MACEs (HR 1.34, 95% CI: 1.08–1.62; *p* < 0.001). Individuals in the high-BNP group (≥300 pg/mL) had a pronounced increase in MACE risk (HR 1.39, 95% CI: 1.10–1.75; *p* < 0.001).

### 3.3 Combined Prognostic Value of Metabolic Indices and BNP

On the basis of the established cutoff values, 1177 patients were stratified into four groups according to their TyG index (<7.2 vs. ≥7.2) and BNP level (<300 vs. ≥300 pg/mL) (Table 1). Patients with both an elevated TyG index and elevated BNP level demonstrated the highest cardiovascular risk profile, characterized by advanced age (median 69.0 years), a greater female proportion (35.3%), a higher diabetes incidence (54.2%), more complex coronary anatomy (elevated SYNTAX scores), and impaired cardiac function (reduced LVEF). Conversely, patients with low TyG index and BNP levels exhibited the most favorable baseline characteristics. Significant between-group differences were observed for most parameters (*p* < 0.001 for age, sex, heart rate, blood pressure, diabetes status, cardiac biomarkers, and medication usage), while smoking status and stroke history did not significantly differ, indicating a clear gradient of cardiovascular risk across biomarker-defined groups.

**Table 1.** **Comparison of baseline characteristics by TyG index and BNP grouping**.

		TyG index <7.2		TyG index ≥7.2		*p* value
		BNP <300, n = 342	BNP ≥300, n = 99	BNP <300, n = 583	BNP ≥300, n = 153	
Age (years)		66.0 (57.0, 72.0)	70.0 (61.0, 76.0)	64.0 (54.0, 70.0)	69.0 (62.0, 75.0)	<0.001
Female, n (%)		63 (18.4%)	26 (26.3%)	122 (20.9%)	54 (35.3%)	<0.001
Heart rate (bpm)		76.0 (67.0, 88.0)	78.0 (70.0, 94.0)	78.0 (68.0, 89.0)	84.0 (73.0, 96.0)	<0.001
SBP (mmHg)		136.0 (122.0, 151.0)	132.0 (117.0, 148.0)	140.0 (124.0, 157.0)	130.0 (113.0, 146.0)	<0.001
DBP (mmHg)		85.0 (75.0, 94.0)	77.0 (68.0, 89.0)	86.0 (77.0, 97.0)	81.0 (71.0, 90.0)	<0.001
Current smoking, n (%)		174 (50.9%)	40 (40.4%)	272 (46.7%)	63 (41.2%)	0.120
Hypertension, n (%)		210 (61.4%)	59 (59.6%)	423 (72.6%)	106 (69.3%)	0.001
Diabetes, n (%)		41 (12.0%)	20 (20.2%)	204 (35.0%)	83 (54.2%)	<0.001
Stroke, n (%)		46 (13.5%)	15 (15.2%)	70 (12.0%)	28 (18.3%)	0.220
Interventions						
Stent, n (%)						0.008
	0	28 (8.2%)	10 (10.1%)	53 (9.1%)	14 (9.2%)	
	1	208 (60.8%)	49 (49.5%)	355 (60.9%)	70 (45.8%)	
	≥2	106 (31.0%)	40 (40.4%)	175 (30.0%)	69 (45.1%)	
SYNTAX score		18.5 (11.0, 22.5)	19.0 (13.0, 25.0)	18.0 (13.0, 24.0)	20.5 (14.5, 28.0)	0.001
rSS		5.0 (2.0, 10.0)	8.0 (3.0, 13.0)	7.0 (2.0, 11.0)	8.0 (3.0, 13.0)	<0.001
LVEF (%)		49.0 (43.0, 56.0)	45.0 (40.0, 50.0)	48.0 (43.0, 55.0)	42.0 (36.0, 47.0)	<0.001
Platelet (×10^9^/L)		219.0 (190.0, 255.0)	217.0 (172.0, 280.0)	224.0 (189.0, 269.0)	224.0 (184.0, 265.0)	0.586
Hemoglobin (g/L)		144.0 (134.0, 156.0)	129.0 (112.0, 141.0)	148.0 (135.0, 159.0)	133.0 (121.0, 148.0)	<0.001
eGFR (mL/min/1.73 m^2^)		100.7 (85.3, 117.2)	93.8 (71.3, 112.6)	97.0 (79.6, 115.4)	83.6 (64.1, 104.9)	<0.001
LDL-C (mmol/L)		2.7 (2.2, 3.2)	2.5 (2.0, 3.0)	3.2 (2.6, 3.8)	3.1 (2.5, 3.8)	<0.001
TnT (ng/mL)		0.2 (0.1, 1.4)	1.4 (0.2, 3.1)	0.2 (0.0, 1.3)	1.2 (0.4, 3.1)	<0.001
TyG index		7.0 (6.7, 7.1)	7.0 (6.7, 7.1)	7.7 (7.5, 8.1)	7.7 (7.5, 8.0)	<0.001
BNP (pg/mL)		54.7 (21.0, 125.0)	553.0 (427.0, 978.0)	48.2 (17.0, 103.0)	669.0 (465.0, 1028.0)	<0.001
P2Y12i, n (%)						<0.001
	Clopidogrel	122 (35.7%)	52 (52.5%)	191 (32.8%)	81 (52.9%)	
	Ticagrelor	220 (64.3%)	47 (47.5%)	392 (67.2%)	72 (47.1%)	
Statin, n (%)						0.002
	Rosuvastatin	313 (91.5%)	78 (78.8%)	525 (90.1%)	140 (91.5%)	
	Atorvastatin	29 (8.5%)	21 (21.2%)	58 (9.9%)	13 (8.5%)	
ACEI/ARB/ARNI, n (%)		90 (26.3%)	28 (28.3%)	216 (37.0%)	49 (32.0%)	0.007
Beta blocker, n (%)		195 (57.0%)	49 (49.5%)	376 (64.5%)	82 (53.6%)	0.004
PCSK9i, n (%)		43 (12.6%)	11 (11.1%)	106 (18.2%)	25 (16.3%)	0.077
SGLT2i, n (%)		27 (7.9%)	15 (15.2%)	136 (23.3%)	49 (32.0%)	<0.001

Abbreviations: SBP, systolic blood pressure; DBP, diastolic blood pressure; SYNTAX, Synergy Between PCI With TAXUS and Cardiac Surgery score; rSS, residual SYNTAX score; LVEF, left ventricular ejection fraction; eGFR, estimated glomerular filtration rate; TNT, troponin T; BNP, B-type natriuretic peptide; LDL-C, low-density lipoprotein cholesterol; P2Y12i, P2Y12 receptor inhibitor; ACEI, angiotensin-converting enzyme inhibitor; ARB, angiotensin receptor blocker; ARNI, angiotensin receptor and neprilysin inhibitor; PCSK9i, PCSK9 inhibitors; SGLT2i, sodium-glucose cotransporter 2 inhibitor.

Similarly, patients were stratified on the basis of TyG–BMI (<186 vs. ≥186) and BNP (<300 vs. ≥300 pg/mL) levels (Table 2). Patients with elevated TyG–BMI and BNP demonstrated the highest cardiovascular risk profile, characterized by older age, higher diabetes incidence (56.5% vs. 18.8% in the low TyG–BMI/low BNP group), more complex coronary disease (higher SYNTAX and residual SYNTAX scores), and reduced LVEF (median 43.0% vs. 48.0%). Patients with a low TyG–BMI and low BNP level exhibited the most favorable characteristics, including younger age (median 67.0 years), lower diabetes incidence (18.8%), and better cardiac function. Significant between-group differences were observed for most clinical parameters (*p* < 0.001 for age, sex, hemodynamics, diabetes status, renal function, and cardiac biomarkers), demonstrating a clear gradient of metabolic and cardiovascular risk across TyG–BMI and BNP-defined strata.

**Table 2.** **Comparison of baseline characteristics by TyG–BMI and BNP grouping**.

		TyG–BMI <186		TyG–BMI ≥186		*p* value
		BNP <300, n = 430	BNP ≥300, n = 144	BNP <300, n = 495	BNP ≥300, n = 108	
Age (years)		67.0 (58.0, 73.0)	70.0 (62.0, 76.0)	62.0 (51.0, 69.0)	68.0 (59.5, 74.5)	<0.001
Female, n (%)		100 (23.3%)	45 (31.2%)	85 (17.2%)	35 (32.4%)	<0.001
Heart rate (bpm)		75.0 (65.0, 86.0)	83.0 (70.0, 95.5)	80.0 (70.0, 90.0)	81.0 (73.0, 95.0)	<0.001
SBP (mmHg)		135.0 (119.0, 151.0)	129.0 (113.5, 146.5)	143.0 (126.0, 157.0)	132.5 (113.5, 151.5)	<0.001
DBP (mmHg)		83.0 (73.0, 93.0)	78.0 (69.5, 89.0)	89.0 (78.0, 98.0)	81.0 (70.0, 92.5)	<0.001
Current smoking, n (%)		198 (46.0%)	57 (39.6%)	248 (50.1%)	46 (42.6%)	0.110
Hypertension, n (%)		266 (61.9%)	89 (61.8%)	367 (74.1%)	76 (70.4%)	<0.001
Diabetes, n (%)		81 (18.8%)	42 (29.2%)	164 (33.1%)	61 (56.5%)	<0.001
Stroke, n (%)		49 (11.4%)	27 (18.8%)	67 (13.5%)	16 (14.8%)	0.158
Interventions						
Stent, n (%)						0.004
	0	32 (7.4%)	15 (10.4%)	49 (9.9%)	9 (8.3%)	
	1	268 (62.3%)	69 (47.9%)	295 (59.6%)	50 (46.3%)	
	≥2	130 (30.2%)	60 (41.7%)	151 (30.5%)	49 (45.4%)	
SYNTAX score		18.0 (12.0, 22.5)	19.0 (13.0, 25.8)	18.5 (12.5, 24.5)	20.2 (15.0, 27.5)	0.003
rSS		5.0 (2.0, 11.0)	8.0 (3.0, 12.0)	7.0 (2.0, 11.0)	9.0 (4.5, 14.0)	<0.001
LVEF (%)		48.0 (43.0, 55.0)	42.0 (38.0, 48.0)	48.0 (43.0, 55.0)	43.0 (38.0, 48.5)	<0.001
Platelet (×10^9^/L)		218.0 (189.0, 256.0)	222.5 (172.5, 280.5)	225.0 (191.0, 268.0)	223.0 (182.5, 257.0)	0.549
Hemoglobin (g/L)		143.0 (131.0, 154.0)	129.5 (116.0, 141.5)	150.0 (139.0, 161.0)	133.0 (120.0, 148.5)	<0.001
eGFR (mL/min/1.73 m^2^)		99.2 (83.3, 114.5)	93.3 (75.0, 113.4)	97.6 (81.2, 118.2)	77.2 (59.0, 98.3)	<0.001
LDL-C (mmol/L)		2.8 (2.3, 3.4)	2.7 (2.0, 3.3)	3.1 (2.6, 3.9)	3.1 (2.5, 3.9)	<0.001
TnT (ng/mL)		0.2 (0.0, 1.3)	1.1 (0.3, 3.1)	0.2 (0.1, 1.4)	1.4 (0.4, 3.1)	<0.001
TyG–BMI index		167.7 (154.3, 177.5)	164.4 (148.3, 173.9)	211.6 (196.0, 234.2)	206.5 (196.1, 223.1)	<0.001
BNP (pg/mL)		57.6 (21.7, 123.0)	602.0 (441.0, 1027.5)	42.0 (16.0, 99.7)	636.5 (457.5, 959.5)	<0.001
P2Y12i, n (%)						<0.001
	Clopidogrel	169 (39.3%)	79 (54.9%)	144 (29.1%)	54 (50.0%)	
	Ticagrelor	261 (60.7%)	65 (45.1%)	351 (70.9%)	54 (50.0%)	
Statin, n (%)						0.064
	Rosuvastatin	390 (90.7%)	120 (83.3%)	448 (90.5%)	98 (90.7%)	
	Atorvastatin	40 (9.3%)	24 (16.7%)	47 (9.5%)	10 (9.3%)	
ACEI/ARB/ARNI, n (%)		120 (27.9%)	32 (22.2%)	186 (37.6%)	45 (41.7%)	<0.001
Beta blocker, n (%)		235 (54.7%)	74 (51.4%)	336 (67.9%)	57 (52.8%)	<0.001
PCSK9i, n (%)		59 (13.7%)	11 (7.6%)	90 (18.2%)	25 (23.1%)	0.002
SGLT2i, n (%)		46 (10.7%)	28 (19.4%)	117 (23.6%)	36 (33.3%)	<0.001

Abbreviations: SBP, systolic blood pressure; DBP, diastolic blood pressure; SYNTAX, SYNTAX score; rSS, residual SYNTAX score; LVEF, left ventricular ejection fraction; eGFR, estimated glomerular filtration rate; TNT, troponin T; BNP, B-type natriuretic peptide; LDL-C, low-density lipoprotein cholesterol; P2Y12i, P2Y12 receptor inhibitor; ACEI, angiotensin-converting enzyme inhibitor; ARB, angiotensin receptor blocker; ARNI, angiotensin receptor and neprilysin inhibitor; PCSK9i, PCSK9 inhibitors; SGLT2i, sodium-glucose cotransporter 2 inhibitor.

### 3.4 Multivariable Cox Regression Analysis

**Fig. 2.**
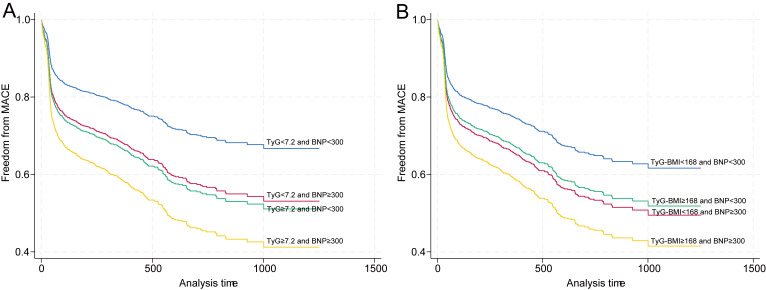
**Synergistic effects of the combined TyG index, TyG–BMI, and BNP stratification on MACE incidence**. (A) Kaplan‒Meier curves for four patient subgroups: low TyG index-low BNP (reference), low TyG index-high BNP (HR 1.57, 95% CI: 1.07–2.30), high TyG index-low BNP (HR 1.66, 95% CI: 1.28–2.14), and high TyG index-high BNP (HR 2.18, 95% CI: 1.57–3.03). (B) Kaplan‒Meier curves for four patient subgroups: low TyG–BMI-low BNP (reference), low TyG–BMI-high BNP (HR 1.45, 95% CI: 1.06–1.99), high TyG–BMI-low BNP (HR 1.35, 95% CI: 1.08–1.71), and high TyG–BMI-high BNP (HR 1.81, 95% CI: 1.30–2.51). Shaded areas around the survival curves represent 95% confidence intervals.

The synergistic effects of the combined TyG index and BNP level on adverse cardiovascular outcomes across the five progressively adjusted models are shown in Table 3 and Fig. 2A. In the unadjusted analysis (Model 1), compared with the reference group (low TyG index-low BNP level), all three groups with at least one elevated biomarker exhibited a significantly higher risk. Following sequential adjustments for demographic characteristics, clinical parameters, procedural variables, and pharmacological interventions, elevated risk persisted across all groups.

**Table 3.** **Effects of the TyG index, TyG–BMI, and BNP levels on outcomes across various models**.

Index type	Model	Hazard ratio (95% CI)				*p* for trend
		Low index + BNP <300	Low index + BNP ≥300	High index + BNP <300	High index + BNP ≥300	
TyG index (cutoff: 7.2)	Model 1	Ref.	2.13 (1.49–3.04)***	1.82 (1.43–2.32)***	3.35 (2.51–4.48)***	<0.001
Sample size:	Model 2	Ref.	2.02 (1.41–2.38)***	1.86 (1.46–2.38)***	3.25 (2.43–4.36)***	<0.001
TyG <7.2 + BNP <300 (n = 342)	Model 3	Ref.	1.80 (1.25–2.60)**	1.65 (1.29–2.11)***	2.38 (1.73–3.28)***	<0.001
TyG <7.2 + BNP ≥300 (n = 99)	Model 4	Ref.	1.57 (1.07–2.30)*	1.66 (1.28–2.14)***	2.18 (1.57–3.03)***	<0.001
TyG ≥7.2 + BNP <300 (n = 583)	Model 5	Ref.	1.57 (1.03–2.39)*	1.66 (1.28–2.15)***	2.18 (1.53–3.12)***	<0.001
TyG ≥7.2 + BNP ≥300 (n = 153)	Model 6	Ref.	2.15 (1.21–3.83)**	1.63 (1.22–2.19)**	2.47 (1.52–4.01)***	0.219
TyG–BMI (cutoff: 186)	Model 1	Ref.	1.84 (1.38–2.46)***	1.42 (1.14–1.76)***	3.01 (2.26–4.02)***	<0.001
Sample size:	Model 2	Ref.	1.76 (1.31–2.35)***	1.52 (1.22–1.90)***	2.99 (2.24–3.99)***	<0.001
TyG–BMI <186 + BNP <300 (n = 430)	Model 3	Ref.	1.55 (1.14–2.11)**	1.39 (1.11–1.74)**	2.14 (1.57–2.92)***	<0.001
TyG–BMI <186 + BNP ≥300 (n = 144)	Model 4	Ref.	1.45 (1.06–1.99)*	1.35 (1.08–1.71)*	1.81 (1.30–2.51)***	0.001
TyG–BMI ≥186 + BNP <300 (n = 495)	Model 5	Ref.	1.45 (1.03–2.05)*	1.35 (1.07–1.72)*	1.81 (1.26–2.60)**	0.001
TyG–BMI ≥186 + BNP ≥300 (n = 108)	Model 6	Ref.	1.63 (1.04–2.57)*	1.25 (0.96–1.62)	1.77 (1.11–2.82)*	0.292

Abbreviations: Model 1 is considered the unadjusted model. Model 2 is adjusted for sex and age. Model 3 includes the variables of Model 2 with the addition of heart rate, SBP, DBP, current smoking status, hypertension status, diabetes status, stroke status, LVEF, SYNTAX score, and rSS. Model 4 builds upon Model 3 by further adjusting for the number of stents, antiplatelet therapy, statin, beta-blockers, ACEI/ARBs/ARNI, PCSK9i, SGLT2i, hemoglobin, platelet count, eGFR, TnT, and LDL-C. Model 5 is an extension of Model 4 with the addition of bootstrapping for statistical robustness. Model 6 is the propensity score matching (PSM) model. * denotes *p* < 0.05, ** denotes *p* < 0.01, and *** denotes *p* < 0.001.

According to the fully adjusted model (Model 4), patients with a low TyG index but elevated BNP levels had a 57% increased risk (HR 1.57, 95% CI: 1.07–2.30), whereas those with a high TyG index and normal BNP levels had a 66% increased risk (HR 1.66, 95% CI: 1.28–2.14). Most notably, patients with both an elevated TyG index and elevated BNP levels presented the highest risk, with a more than twofold increase (HR 2.18, 95% CI: 1.57–3.03). Bootstrap analysis (Model 5) validated the robustness of these findings, with consistent hazard ratios and maintained statistical significance. Additionally, the results of the propensity score matching analysis (Model 6) revealed a good balance in baseline characteristics across groups (**Supplementary Tables 4,5**). The results remained consistent with those of the multivariable-adjusted models, with the dual-high group (TyG index ≥7.2 and BNP ≥300) showing the highest risk (HR 2.47, 95% CI: 1.52–4.01). This further reinforces the robustness and persuasiveness of our conclusions.

TyG–BMI stratification revealed comparable risk elevation patterns (Table 3 and Fig. 2B). In the fully adjusted model (Model 4), patients with low TyG–BMI but elevated BNP levels had a 45% increased risk (HR 1.45, 95% CI: 1.06–1.99), whereas those with high TyG–BMI and normal BNP levels had a 35% increased risk (HR 1.35, 95% CI: 1.08–1.71). The dual high biomarker group exhibited an 81% increased risk (HR 1.81, 95% CI: 1.30–2.51). Bootstrap validation (Model 5) confirmed the association stability, with significant dose‒response relationships (*p* for trend < 0.001) maintained across all model iterations, establishing the independent prognostic value of combined metabolic and cardiac biomarker assessment.

Consistently, PSM analysis (Model 6) yielded similar results, with the dual-high group (TyG–BMI ≥186 and BNP ≥300) demonstrating the highest risk (HR 1.77, 95% CI: 1.11–2.82), further supporting the robustness of these associations.

### 3.5 Subgroup Analysis

Subgroup analysis (Table 4) revealed significant interactions between metabolic indices and BNP levels across various patient populations. With respect to the TyG index (cutoff: 7.2), compared with the reference group (low TyG + BNP level <300), patients with a high TyG index and elevated BNP level (≥300 pg/mL) had markedly increased hazard ratios. This association was particularly pronounced in elderly patients (≥65 years), those with an HR of 3.09 (95% CI: 2.07–4.60; *p* < 0.01), female patients (HR 2.90; 95% CI: 1.49–5.65; *p* = 0.004), patients with hypertension (HR 2.34; 95% CI: 1.59–3.44; *p* < 0.01), nondiabetic patients (HR 2.78; 95% CI: 1.85–4.18; *p* < 0.01), and those with low SYNTAX scores <22 (HR 3.12; 95% CI: 1.90–5.12; *p* < 0.01).

**Table 4.** **Subgroup analysis of the TyG index and TyG–BMI with BNP levels**.

Index type	Subgroup	Hazard ratio (95% CI)				*p* for trend
		Low index + BNP <300	Low index + BNP ≥300	High index + BNP <300	High index + BNP ≥300	
TyG index (cutoff: 7.2)	Age ≥65					
Sample size:	No	Ref.	1.31 (0.63–2.75)	1.77 (1.19–2.65)	1.13 (0.62–2.08)	0.066
TyG <7.2 + BNP <300 (n = 342)	Yes	Ref.	1.69 (1.06–2.72)	1.51 (1.07–2.13)	3.09 (2.07–4.60)	<0.001
TyG <7.2 + BNP ≥300 (n = 99)	Female					
TyG ≥7.2 + BNP <300 (n = 583)	No	Ref.	1.69 (1.09–2.63)	1.74 (1.31–2.33)	2.1 (1.41–3.11)	<0.001
TyG ≥7.2 + BNP ≥300 (n = 153)	Yes	Ref.	1.61 (0.72–3.60)	1.59 (0.89–2.84)	2.9 (1.49–5.65)	0.004
	Hypertension					
	No	Ref.	1.49 (0.76–2.92)	1.34 (0.82–2.19)	1.5 (0.76–2.98)	0.227
	Yes	Ref.	1.5 (0.92–2.42)	1.74 (1.28–2.35)	2.34 (1.59–3.44)	<0.001
	Diabetes					
	No	Ref.	1.22 (0.76–1.94)	1.77 (1.32–2.36)	2.78 (1.85–4.18)	<0.001
	Yes	Ref.	2.79 (1.33–5.86)	1.29 (0.74–2.28)	1.68 (0.89–3.18)	0.343
	SYNTAX Score ≥22					
	No	Ref.	1.75 (1.00–3.06)	1.99 (1.36–2.90)	3.12 (1.90–5.12)	<0.001
	Yes	Ref.	1.38 (0.79–2.40)	1.37 (0.96–1.94)	1.81 (1.16–2.83)	0.013
	SGLT2i					
	No	Ref.	1.39 (0.90–2.14)	1.66 (1.25–2.19)	2.15 (1.47–3.14)	<0.001
	Yes	Ref.	2.05 (0.84–5.02)	0.95 (0.48–1.88)	1.46 (0.67–3.21)	0.642
TyG–BMI (cutoff: 186)	Age ≥65					
Sample size:	No	Ref.	1.03 (0.54–1.95)	1.37 (0.96–1.95)	0.87 (0.47–1.60)	0.344
TyG–BMI <186 + BNP <300 (n = 430)	Yes	Ref.	1.64 (1.13–2.39)	1.25 (0.92–1.71)	2.98 (2.01–4.41)	<0.001
TyG–BMI <186 + BNP ≥300 (n = 144)	Female					
TyG–BMI ≥186 + BNP <300 (n = 495)	No	Ref.	1.63 (1.12–2.36)	1.45 (1.11–1.89)	1.64 (1.09–2.48)	0.004
TyG–BMI ≥186 + BNP ≥300 (n = 108)	Yes	Ref.	1.23 (0.64–2.34)	1.07 (0.65–1.76)	2.48 (1.37–4.51)	0.032
	Hypertension					
	No	Ref.	1.64 (0.90–2.98)	1.23 (0.76–1.98)	1.08 (0.54–2.16)	0.632
	Yes	Ref.	1.31 (0.89–1.91)	1.33 (1.01–1.74)	1.99 (1.36–2.92)	0.001
	Diabetes					
	No	Ref.	1.33 (0.91–1.96)	1.46 (1.10–1.93)	2.23 (1.42–3.49)	<0.001
	Yes	Ref.	1.5 (0.85–2.65)	1.05 (0.69–1.62)	1.63 (0.95–2.79)	0.281
	SYNTAX Score ≥22					
	No	Ref.	1.76 (1.12–2.76)	1.5 (1.08–2.09)	1.98 (1.24–3.17)	0.003
	Yes	Ref.	1.26 (0.79–1.98)	1.17 (0.84–1.63)	1.78 (1.10–2.88)	0.066
	SGLT2i					
	No	Ref.	1.42 (1.00–2.02)	1.24 (0.96–1.61)	1.47 (0.99–2.19)	0.043
	Yes	Ref.	1.51 (0.71–3.25)	1.43 (0.79–2.59)	2.76 (1.36–5.60)	0.015

Abbreviations: BNP, B-type natriuretic peptide; CI, confidence interval; SGLT2i, sodium-glucose cotransporter 2 inhibitor; TyG, triglyceride–glucose index; TyG–BMI, triglyceride–glucose–body mass index. Notes: All analyses were performed using the fully adjusted model (Model 5 from Table 3). *p*-values < 0.05 were considered to indicate statistical significance.

Similarly, TyG–BMI (cutoff: 186) showed consistent patterns, with the highest risk observed in elderly patients with high TyG–BMI and elevated BNP (HR 2.98; 95% CI: 2.01–4.41; *p* < 0.01) and nondiabetic patients (HR 2.23; 95% CI: 1.42–3.49; *p* < 0.01). Notably, the combination of high metabolic indices with elevated BNP consistently yielded the strongest predictive associations across most subgroups, suggesting synergistic effects between metabolic dysfunction and cardiac stress markers in risk stratification.

These findings indicate that the prognostic value of the TyG and TyG–BMI indices is significantly enhanced when these indices are combined with BNP levels, particularly in high-risk populations, including elderly, female, hypertensive, and complex coronary disease patients.

To provide deeper clinical insight, we evaluated the hazard ratios for individual MACE components within these subgroups (Tables 5,6). Patients with elevated levels of both metabolic markers and BNP had significantly greater risks of adverse outcomes, including all-cause mortality, nonfatal myocardial infarction, cerebrovascular events, heart failure hospitalization, and ischemia-induced revascularization, further emphasizing the prognostic utility of combining TyG indices with BNP in high-risk patients.

**Table 5.** **Hazard ratios (95% CI) for individual MACE components stratified by TyG index and BNP levels**.

Outcome	TyG index <7.2		TyG index ≥7.2	
	BNP <300, n = 342	BNP ≥300, n = 99	BNP <300, n = 583	BNP ≥300, n = 153
All-cause mortality	Ref.	0.99 (0.19–5.28)	1.16 (0.36–3.75)	4.32 (1.30–14.4)
Nonfatal myocardial infarction	Ref.	4.56 (1.34–15.5)*	2.26 (0.95–5.37)	3.03 (0.94–9.72)
Cerebrovascular event	Ref.	1.20 (0.24–5.99)	1.69 (0.72–3.99)	3.74 (1.12–12.5)*
Heart failure hospitalization	Ref.	0.74 (0.24–2.30)	2.04 (1.00–4.17)*	2.29 (0.99–5.26)
Ischemia-induced revascularization	Ref.	1.69 (1.03–2.78)*	1.53 (1.11–2.11)**	1.52 (0.95–2.44)

Abbreviations: BNP, B-type natriuretic peptide; CI, confidence interval; TyG, triglyceride–glucose index. *p < 0.05, **p < 0.01.

**Table 6.** **Hazard ratios (95% CI) for individual MACE components stratified by TyG–BMI and BNP levels**.

Outcome	TyG–BMI <186		TyG–BMI ≥186	
	BNP <300, n = 430	BNP ≥300, n = 144	BNP <300, n = 495	BNP ≥300, n = 108
All-cause mortality	Ref.	2.39 (0.81–7.06)	0.79 (0.28–2.17)	2.74 (0.91–8.21)
Nonfatal myocardial infarction	Ref.	2.85 (1.06–7.62)*	1.13 (0.56–2.27)	1.32 (0.41–4.29)
Cerebrovascular event	Ref.	0.79 (0.17–3.75)	1.57 (0.71–3.47)	5.02 (1.63–15.5)**
Heart failure hospitalization	Ref.	1.23 (0.55–2.74)	2.69 (1.37–5.27)**	2.64 (1.14–6.07)*
Ischemia-induced revascularization	Ref.	1.27 (0.81–1.99)	1.32 (0.98–1.77)	1.50 (0.95–2.37)

Abbreviations: BNP, B-type natriuretic peptide; CI, confidence interval; TyG–BMI, triglyceride–glucose–body mass index. *p < 0.05, **p < 0.01.

### 3.6 Analysis of Receiver Operating Characteristics

**Fig. 3.**
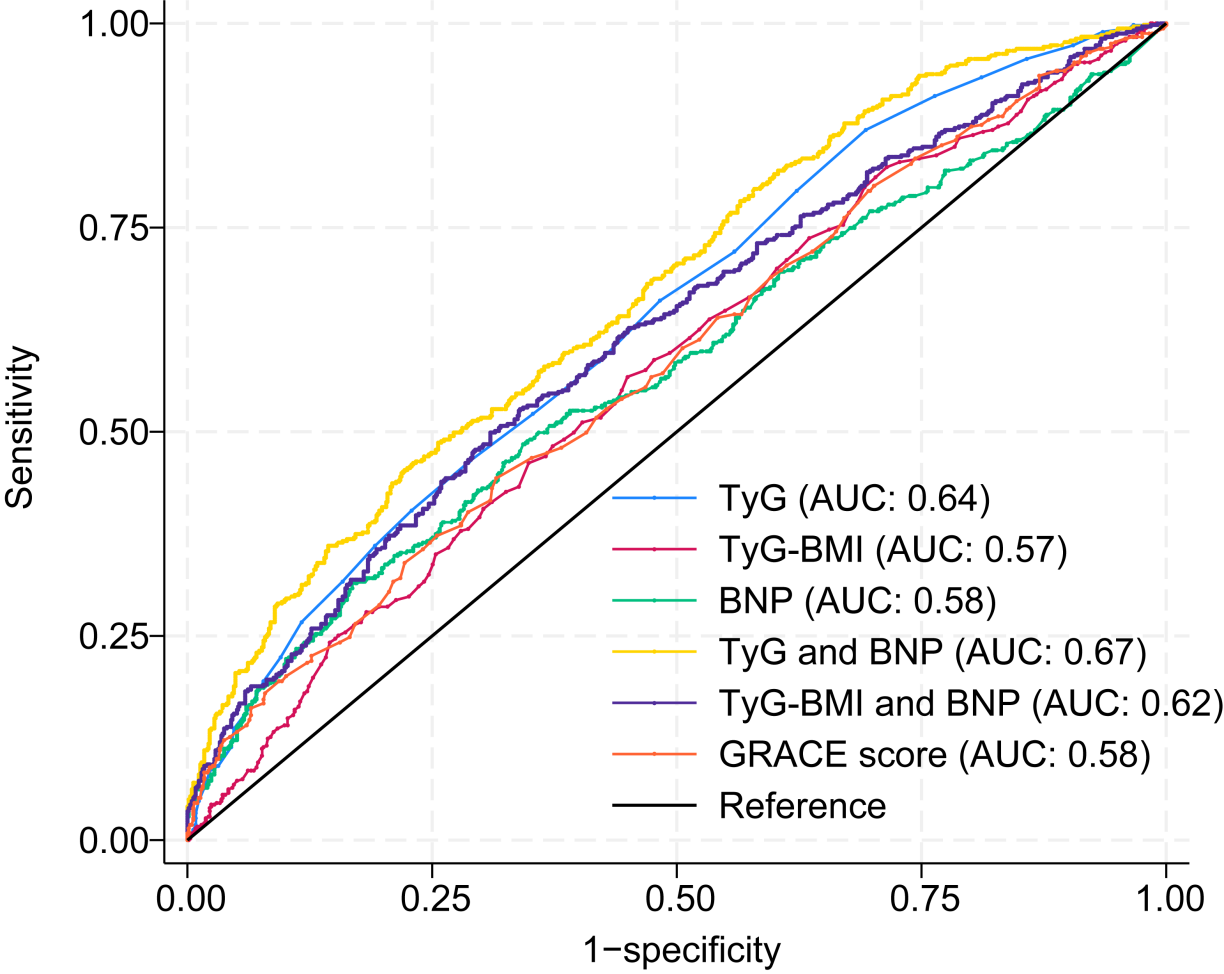
**Discriminatory capacity of individual and combined biomarker models for MACE prediction**. Receiver operating characteristic curves comparing the prognostic performance of the TyG index alone (AUC: 0.64, 95% CI: 0.60–0.67; *p* < 0.001), the TyG–BMI alone (AUC: 0.57, 95% CI: 0.54–0.60; *p* < 0.001), the BNP alone (AUC: 0.58, 95% CI: 0.54–0.61; *p* < 0.001), the GRACE score (AUC: 0.58, 95% CI: 0.56–0.62; *p* < 0.001), and the integrated TyG index + BNP model (AUC: 0.67, 95% CI: 0.64–0.70; *p* < 0.001). GRACE, Global Registry of Acute Coronary Events; AUC, area under the curve.

To evaluate the incremental predictive value of biomarker integration, receiver operating characteristic analysis was performed to compare the discriminatory performance of the combined models (TyG index + BNP and TyG–BMI + BNP) against that of the individual biomarkers and the established GRACE score. The integrated TyG index + BNP model demonstrated superior predictive accuracy, with an AUC of 0.67 (95% CI: 0.64–0.70; *p* < 0.001; Fig. 3), outperforming both the TyG–BMI + BNP combination (AUC: 0.62, 95% CI: 0.59–0.65; *p* < 0.001) and the conventional GRACE score (AUC: 0.58, 95% CI: 0.56–0.62; *p* < 0.001), as well as each individual biomarker when evaluated separately. This enhanced discriminatory capacity supports the clinical utility of combined metabolic and cardiac biomarker assessment for MACE prediction in STEMI patients.

In addition, sensitivity and specificity analyses were performed to further evaluate the discriminatory performance of each indicator (Table 7). The results revealed that the TyG index, TyG–BMI, BNP level, TyG + BNP level, TyG–BMI + BNP level, and GRACE score had sensitivities of 66%, 57%, 53%, 58%, 53%, and 47%, respectively, and specificities of 52%, 55%, 61%, 64%, 66%, and 65%, respectively. These findings further support the enhanced predictive value of combined biomarker models beyond conventional risk scores.

**Table 7.** **Discriminatory performance of biomarkers and the risk score for predicting MACEs in STEMI patients**.

Model	AUC	Sensitivity, %	Specificity, %
TyG index	0.64, 95% CI: 0.60–0.67	66	52
TyG–BMI	0.57, 95% CI: 0.54–0.60	57	55
BNP	0.58, 95% CI: 0.54–0.61	53	61
TyG index and BNP	0.67, 95% CI: 0.64–0.70	58	64
TyG–BMI and BNP	0.62, 95% CI: 0.59–0.65	53	66
GRACE score	0.58, 95% CI: 0.56–0.62	47	65

Abbreviations: BNP, B-type natriuretic peptide; CI, confidence interval; TyG, triglyceride–glucose index; TyG–BMI, triglyceride–glucose–body mass index.

The predictive efficacy of the TyG index and TyG–BMI combined with BNP was further evaluated across different subgroups. The results demonstrated enhanced predictive performance in specific populations. In elderly patients (age ≥65 years), the AUCs for the TyG index and TyG–BMI combined with BNP were 0.68 and 0.65, respectively. In female patients, the AUCs for the TyG index and TyG–BMI combined with BNP were 0.67 and 0.65, respectively. In hypertensive patients, the AUCs for TyG and TyG–BMI combined with BNP were 0.68 and 0.61, respectively. However, predictive efficacy was weaker in patients with diabetes or those receiving SGLT2i therapy, with lower AUC values. These findings suggest that the predictive value of TyG-related indices is more pronounced in specific subgroups than in the overall population. Full details of the subgroup analyses are provided in Table 8.

**Table 8.** **Subgroup predictive efficacy of the TyG index and TyG–BMI combined with BNP**.

Subgroup		AUC (95% CI)	
		TyG index and BNP	TyG–BMI and BNP
Age ≥65			
	No	0.66 (0.62, 0.71)	0.59 (0.54, 0.64)
	Yes	0.68 (0.64, 0.72)	0.65 (0.61, 0.70)
Female			
	No	0.67 (0.63, 0.70)	0.61 (0.57, 0.65)
	Yes	0.67 (0.61, 0.74)	0.65 (0.58, 0.71)
Hypertension			
	No	0.64 (0.58, 0.70)	0.63 (0.57, 0.69)
	Yes	0.68 (0.64, 0.72)	0.61 (0.57, 0.65)
Diabetes			
	No	0.67 (0.63, 0.70)	0.62 (0.58, 0.66)
	Yes	0.64 (0.58, 0.70)	0.56 (0.50, 0.62)
SYNTAX Score ≥22			
	No	0.69 (0.65, 0.73)	0.62 (0.58, 0.67)
	Yes	0.63 (0.58, 0.68)	0.60 (0.55, 0.66)
SGLT2i			
	No	0.68 (0.65, 0.71)	0.63 (0.59, 0.66)
	Yes	0.58 (0.51, 0.66)	0.54 (0.46, 0.61)

Abbreviations: BNP, B-type natriuretic peptide; CI, confidence interval; TyG, triglyceride–glucose index; TyG–BMI, triglyceride–glucose–body mass index; SGLT2i, sodium-glucose cotransporter 2 inhibitor.

## 4. Discussion

Our comprehensive retrospective cohort study of 1177 STEMI patients provides compelling evidence that the combination of metabolic indices (TyG index or TyG–BMI index) with BNP significantly enhances prognostic stratification for MACE prediction. The key findings demonstrate that patients with elevated levels of both metabolic markers and BNP exhibit substantially increased cardiovascular risk, with hazard ratios exceeding 2.0 for the highest-risk combinations. These results suggest that compared with conventional assessment methods, integrated biomarker approaches may offer superior risk discrimination.

### 4.1 Metabolic Dysfunction and Cardiovascular Risk in STEMI

The strong association between an elevated TyG index and adverse cardiovascular outcomes observed in our study aligns with growing evidence indicating that insulin resistance is a critical determinant of postinfarction prognosis. Insulin resistance promotes endothelial dysfunction, accelerated atherosclerosis, and prothrombotic states through multiple mechanisms, including increased oxidative stress, inflammatory cytokine activation, and altered lipid metabolism [20, 21]. The TyG index, as a simple and readily available surrogate marker of insulin resistance, offers practical advantages over more complex assessments, such as the hyperinsulinemic–euglycemic clamp technique [22]. Similarly, a previous clinical study first reported that higher TyG index values were significantly associated with increased MACE risk in STEMI patients, suggesting its potential as a valid predictor of outcomes after PCI [23]. These findings underscore the prognostic value of the TyG index and its utility in risk stratification following STEMI.

Our findings regarding the TyG–BMI index provide additional insights into the role of adiposity-related metabolic dysfunction in STEMI outcomes. Incorporating BMI into metabolic risk assessments may capture additional pathophysiological dimensions, including adipose tissue dysfunction, systemic inflammation, and altered adipokine profiles, that contribute to cardiovascular risk [24, 25]. The observed linear relationship between the TyG–BMI index and MACE risk, in contrast to the threshold effect seen for the TyG index, suggests potentially different underlying mechanisms and may inform clinical decision-making regarding risk stratification. These findings are consistent with those of Liu *et al*. [9], who likewise demonstrated that higher TyG–BMI values predict adverse outcomes in STEMI patients after PCI, reinforcing its prognostic relevance in the acute infarction setting.

In addition, by comparing integrated biomarker models, we observed that combining the TyG index with BNP level achieved better discriminatory performance than either the TyG–BMI or the conventional GRACE score did, underscoring the value of joint metabolic–cardiac biomarker assessment. Clinically, these results suggest that while the TyG–BMI can identify patients at elevated risk, greater predictive accuracy may be achieved through biomarker integration, which could assist in refining risk stratification and guiding secondary prevention strategies in real-world STEMI management.

### 4.2 Cardiac Stress Response and Prognosis

The prognostic significance of elevated BNP levels in our STEMI cohort corroborates the extensive literature demonstrating the utility of natriuretic peptides for cardiovascular risk assessment. BNP elevation reflects multiple pathophysiological processes, including ventricular dysfunction, increased wall stress, and neurohormonal activation, all of which contribute to adverse cardiovascular outcomes [26, 27]. The threshold effect observed at a BNP level ≥300 pg/mL in our restricted cubic spline analysis provides practical guidance for clinical risk stratification and is broadly consistent with the established cutoff values used in acute heart failure diagnosis and management [28, 29].

### 4.3 Synergistic Effects of Combined Biomarker Assessment

The most significant contribution of our study lies in demonstrating the synergistic prognostic value of combining metabolic and cardiac stress markers. Patients with both an elevated TyG index and elevated BNP levels had a more than twofold increased MACE risk, substantially exceeding the risk associated with either biomarker alone. These findings suggest that metabolic dysfunction and cardiac stress represent complementary pathophysiological domains that, when present simultaneously, confer particularly high cardiovascular risk.

The biological rationale for this synergistic effect may involve several interconnected mechanisms. Insulin resistance can exacerbate cardiac dysfunction through impaired myocardial glucose utilization, increased oxidative stress, and the promotion of myocardial fibrosis [30, 31]. Conversely, cardiac dysfunction may worsen insulin resistance through altered tissue perfusion, neurohormonal activation, and systemic inflammation [21, 32]. This bidirectional relationship may create a pathophysiological cycle that amplifies cardiovascular risk when both conditions coexist.

### 4.4 Clinical Implications and Risk Stratification

The superior discriminatory capacity of the combined biomarker models compared with the established GRACE score (AUC: 0.67 vs. 0.58) has important clinical implications. The GRACE score, while widely validated and recommended by guidelines, primarily incorporates demographic and clinical variables available at presentation [3]. Our findings suggest that the addition of readily available biomarkers may increase the accuracy of risk prediction, potentially enabling more personalized therapeutic approaches.

The subgroup analyses revealed particularly pronounced associations in elderly patients, women, and hypertensive patients, suggesting that certain populations may derive greater benefit from combined biomarker assessment. These findings may inform targeted risk stratification strategies and help identify patients who would benefit from intensive monitoring and aggressive therapeutic interventions.

### 4.5 Therapeutic Implications

The identification of high-risk patients through combined biomarker assessment may guide therapeutic decision-making in several domains. Patients with elevated metabolic indices may benefit from intensive glucose management, lipid-lowering therapy, and lifestyle interventions targeting insulin resistance [20, 33]. Those with elevated BNP levels may require closer monitoring for heart failure development and earlier initiation of guideline-directed medical therapy for left ventricular dysfunction [27].

Furthermore, emerging therapeutic approaches targeting metabolic dysfunction, such as SGLT2 inhibitors and Glucagon-Like Peptide-1 (GLP-1) receptor agonists, have demonstrated cardiovascular benefits in patients with and without diabetes [34, 35]. The identification of high-risk patients through metabolic biomarker assessment may help guide the selection of patients most likely to benefit from these novel therapeutic interventions.

### 4.6 Limitations

Several limitations of our study warrant consideration. First, the retrospective design may introduce selection bias and limit the ability to establish causal relationships. Second, the single-center nature of our study may limit its generalizability to other populations and health care systems. Third, biomarker measurements were obtained at a single time point, and temporal changes in biomarker levels during follow-up were not assessed. Fourth, while we adjusted for multiple confounding variables, residual confounding from unmeasured factors cannot be completely excluded.

Additionally, the follow-up period, while adequate for detecting MACE occurrence, may not capture long-term cardiovascular outcomes. The composite nature of our primary endpoint, while clinically relevant, may obscure differences in individual outcome components. Finally, the cutoff values used for biomarker stratification were derived from our study population and may require validation in independent cohorts.

### 4.7 Future Directions

Our findings provide a foundation for several important research directions. Prospective validation studies in independent STEMI populations are needed to confirm the generalizability of our results. An investigation of optimal biomarker cutoff values across different populations and clinical settings may enhance the clinical utility of combined biomarker assessment.

The development of integrated risk prediction models incorporating multiple biomarker domains, clinical variables, and emerging risk factors represents an important area for future investigations. Additionally, studies examining the cost-effectiveness of biomarker-guided risk stratification strategies will be essential for informing clinical practice guidelines and health care policy decisions.

Finally, interventional studies examining whether biomarker-guided therapeutic approaches improve clinical outcomes compared with standard care would provide definitive evidence for the clinical utility of combined biomarker assessment in STEMI management.

## 5. Conclusions

Our study demonstrated that combining the TyG index or TyG–BMI with BNP level significantly enhanced MACE prediction in STEMI patients, with patients with both elevated metabolic markers and elevated BNP showing a more than twofold increase in cardiovascular risk. Compared with individual biomarkers and established risk scores, the integrated biomarker approach provides superior discriminatory capacity, supporting its potential utility for personalized risk stratification and therapeutic decision-making in clinical practice. Prospective validation studies are warranted to confirm these findings and establish the clinical utility of this combined biomarker strategy.

## Data Availability

The datasets used and analyzed during the current study are available from the corresponding author on reasonable request, subject to appropriate ethical approvals and data sharing agreements.

## Abbreviations

MACE, Major adverse cardiovascular event; STEMI, ST-elevation myocardial infarction; BMI, Body mass index; BNP, B-type natriuretic peptide; DBP, Diastolic blood pressure; GRACE, Global Registry of Acute Coronary Events; LDL-C, Low-density lipoprotein cholesterol; LVEF, Left ventricular ejection fraction; SBP, Systolic blood pressure; eGFR, Estimated glomerular filtration rate; TNT, Troponin T; PCI, Percutaneous coronary intervention; rSS, Residual SYNTAX score; SYNTAX score, SYNergy between PCI with TAXUS and the Cardiac Surgery score; ACEI, Angiotensin-converting enzyme inhibitor; ARB, Angiotensin receptor blocker; ARNI, Angiotensin receptor and neprilysin inhibitor; PCSK9i, Proprotein convertase subtilisin/kexin type 9 inhibitor; SGLT2i, Sodium-glucose cotransporter 2 inhibitor; ROC, Receiver operating characteristic; IQR, Interquartile range; HR, Hazard ratio; CI, Confidence interval; RCSs, Restricted cubic splines.

## Supplementary Material

Supplementary material associated with this article can be found, in the online version, at https://doi.org/10.31083/RCM44062.
