# Supplementary material for: Combined Triglyceride–Glucose and Triglyceride–Glucose–Body Mass Index with B-Type Natriuretic Peptide for Enhanced Prediction of Major Adverse Cardiovascular Events in ST-Elevation Myocardial Infarction Patients: A Retrospective Cohort Study
Source: Rev Cardiovasc Med. 2026 Jan 21;27(1):44062. doi: 10.31083/RCM44062 (PMC12873706; doi:10.31083/RCM44062)
Supplement: Supplementary file 1 [file 2153-8174-27-1-44062-s1.zip › Supplementary Material.docx]

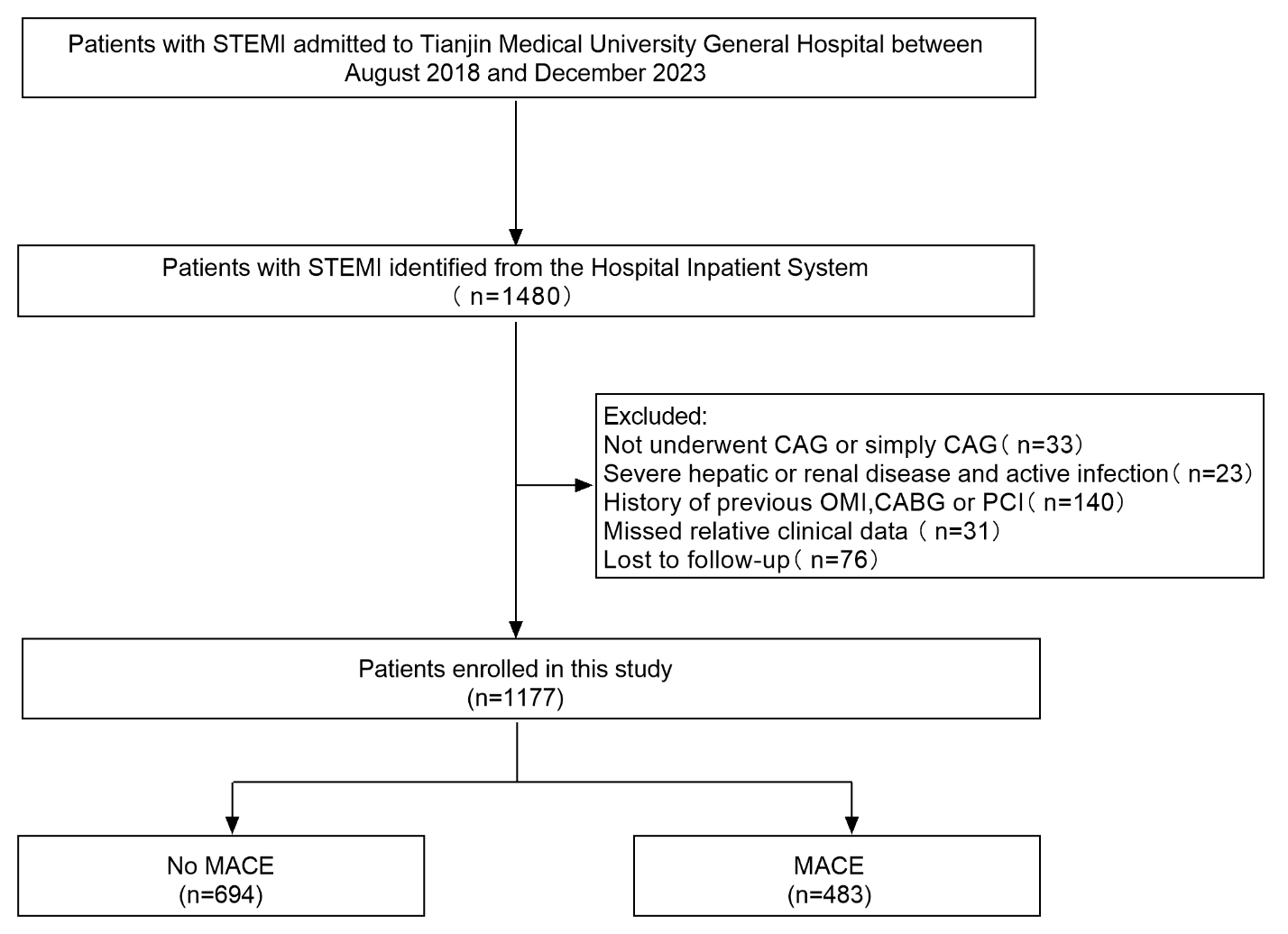


**Supplementary Fig. 1**. Flowchart of the detailed selection process

Abbreviations: STEMI: ST-segment elevation myocardial infarction, CAG: coronary angiography, OMI: old myocardial infarction, CABG: coronary artery bypass graft, PCI: percutaneous coronary intervention, MACE: adverse cardiovascular event.


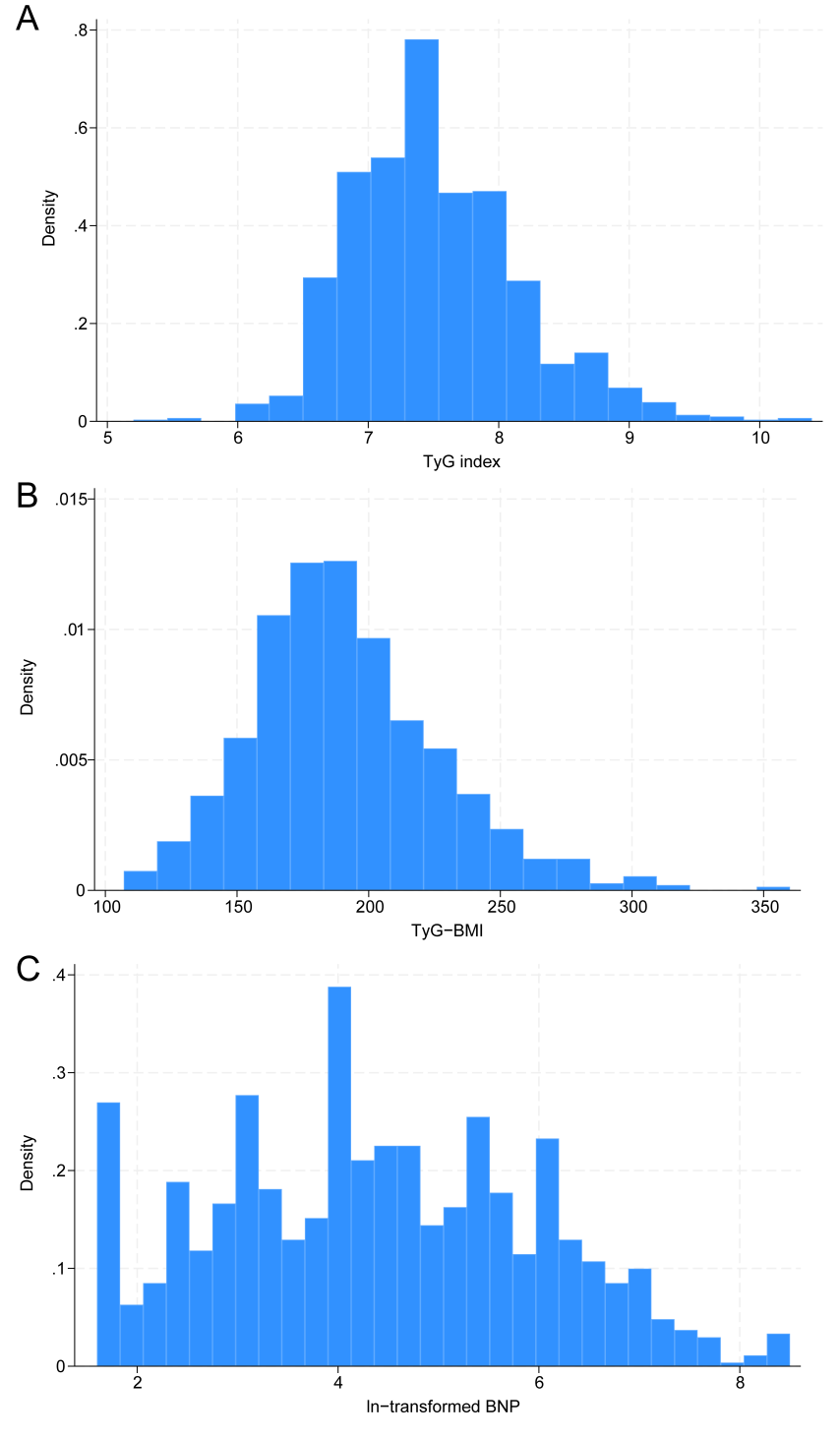


**Supplementary Fig. 2**. Frequency distributions of the TyG index, TyG-BMI, and BNP level

Panel A: histogram of the TyG index; Panel B: histogram of TyG-BMI; Panel C: histogram of the BNP level.

**Supplementary Table 1**. Percentage breakdown of individual MACE components

| MACE | Number (%) |
| --- | --- |
| All-cause mortality | 41 (3.48) |
| Nonfatal myocardial infarction | 53 (4.5) |
| Cerebrovascular event | 37 (3.14) |
| Heart failure hospitalization | 83 (7.05) |
| Ischemia-induced revascularization | 269 (22.9) |

Abbreviations: MACE: adverse cardiovascular event.

**Supplementary Table 2.** Baseline characteristics between patients with and without MACEs

|  | No MACE  n= 694 | MACE  n= 483 | *P* value |
| --- | --- | --- | --- |
| Age (years) | 65 (54, 71) | 67 (59, 73) | \| <0.001 \| \| --- \| |
| Female, n (%) | 145 (20.9%) | 120 (24.8%) | 0.11 |
| Heart rate (bpm) | 78 (69, 89) | 78 (69, 91) | 0.66 |
| SBP (mmHg) | 137 (122, 153) | 138 (120, 154) | 0.63 |
| DBP (mmHg) | 85 (75, 95) | 83 (73, 95) | 0.19 |
| Current smoking, n (%) | 335 (48.3%) | 214 (44.3%) | 0.18 |
| History of disease |  |  |  |
| Hypertension, n (%) | 444 (64.0%) | 354 (73.3%) | <0.001 |
| Diabetes, n (%) | 167 (24.1%) | 181 (37.5%) | <0.001 |
| Stroke, n (%) | 87 (12.5%) | 72 (14.9%) | 0.24 |
| Interventions |  |  |  |
| Number of stents, n (%) |  |  | <0.001 |
| 0 | 59 (8.5%) | 46 (9.5%) |  |
| 1 | 437 (63.0%) | 245 (50.7%) |  |
| ≥2 | 198 (28.5%) | 192 (39.8%) |  |
| SYNTAX score | 16.0 (11.0, 21.5) | 22.0 (17.0, 27.5) | <0.001 |
| rSS | 5.0 (1.0, 8.0) | 10.0 (5.0, 15.0) | <0.001 |
| LVEF (%) | 48.0 (42.0, 55.0) | 47.0 (41.0, 54.0) | 0.004 |
| Laboratory variables |  |  |  |
| Platelet (*10^9^/L) | 223.5 (190.0, 265.0) | 219.0 (185.0, 260.0) | 0.43 |
| Hemoglobin (g/L) | 146.0 (132.0, 156.0) | 141.0 (128.0, 154.0) | 0.004 |
| eGFR (ml/min/1.73㎡) | 99.5 (83.3, 116.8) | 92.2 (71.3, 111.6) | <0.001 |
| LDL-C (mmol/L) | 2.9 (2.4, 3.5) | 3.0 (2.4, 3.7) | 0.19 |
| TNT (ng/mL) | 0.3 (0.1, 1.5) | 0.5 (0.1, 2.1) | 0.003 |
| TyG index | 7.3 (7.0, 7.7) | 7.6 (7.2, 8.1) | <0.001 |
| TyG-BMI | 183.2 (164.5, 206.9) | 191.5 (171.9, 219.7) | <0.001 |
| BNP (pg/mL) | 64.0 (22.0, 206.0) | 103.0 (28.1, 408.0) | <0.001 |
| Discharge medication |  |  |  |
| P2Y12 inhibitor |  |  | 0.002 |
| Clopidogrel, n (%) | 238 (34.3%) | 208 (43.1%) |  |
| Ticagrelor, n (%) | 456 (65.7%) | 275 (56.9%) |  |
| Statin |  |  | 0.044 |
| Rosuvastatin, n (%) | 633 (91.2%) | 423 (87.6%) |  |
| Atorvastatin, n (%) | 61 (8.8%) | 60 (12.4%) |  |
| ACEI/ARB/ARNI, n (%) | 220 (31.7%) | 163 (33.7%) | 0.46 |
| Beta blocker, n (%) | 434 (62.5%) | 268 (55.5%) | 0.015 |
| PCSK9i, n (%) | 94 (13.5%) | 91 (18.8%) | 0.014 |
| SGLT2i, n (%) | 108 (15.6%) | 119 (24.6%) | <0.001 |

Abbreviations: BMI: body mass index, SBP: systolic blood pressure, DBP: diastolic blood pressure, SYNTAX: SYNTAX score, rSS: residual SYNTAX score, LVEF: left ventricular ejection fraction, BNP: B-type natriuretic peptide, LDL-C: low-density lipoprotein cholesterol, ACEI: angiotensin-converting enzyme inhibitor, ARB: angiotensin receptor blocker, ARNI: angiotensin receptor and neprilysin inhibitor, PCSK9i: PCSK9 inhibitor, SGLT2i: sodium–glucose cotransporter 2 inhibitor, MACE: adverse cardiovascular event.

**Supplementary Table 3.** Collinearity diagnostics based on the variance inflation factor (VIF)

| Variable | VIF |
| --- | --- |
| SYNTAX score | 2.63 |
| DBP | 2.47 |
| SBP | 2.45 |
| rSS | 2.39 |
| Hemoglobin | 1.62 |
| Age | 1.60 |
| LVEF | 1.57 |
| Sex | 1.56 |
| Diabetes | 1.48 |
| SGLT2i | 1.42 |
| BNP | 1.39 |
| Heart rate | 1.33 |
| Hypertension | 1.30 |
| Current smoking | 1.26 |
| LDL-C | 1.23 |
| eGFR | 1.23 |
| P2Y12i | 1.21 |
| Beta blocker | 1.19 |
| PCSK9i | 1.15 |
| Platelet | 1.12 |
| ACEI/ARB/ARNI | 1.11 |
| Stent | 1.09 |
| Stroke | 1.08 |
| TNT | 1.07 |
| Statin | 1.05 |

Abbreviations: BMI: body mass index, SBP: systolic blood pressure, DBP: diastolic blood pressure, SYNTAX: SYNTAX score, rSS: residual SYNTAX score, LVEF: left ventricular ejection fraction, BNP: B-type natriuretic peptide, LDL-C: low-density lipoprotein cholesterol, ACEI: angiotensin-converting enzyme inhibitor, ARB: angiotensin receptor blocker, ARNI: angiotensin receptor and neprilysin inhibitor, PCSK9i: PCSK9 inhibitor, SGLT2i: sodium–glucose cotransporter 2 inhibitor.

**Supplementary Table 4.** Baseline Characteristics of Matched Cohorts Using the Low-Level Group as the Reference

|  | *P* value | | | |
| --- | --- | --- | --- | --- |
|  | TyG index< 7.2 and BNP< 300 | TyG index< 7.2 and BNP≥300 | TyG index≥ 7.2 and BNP< 300 | TyG index≥ 7.2 and BNP≥300 |
| Age | *Ref.* | 0.57 | 0.83 | 0.76 |
| Sex | *Ref.* | 0.71 | 1.00 | 0.51 |
| Heart rate | *Ref.* | 0.55 | 0.28 | 0.59 |
| SBP | *Ref.* | 0.55 | 0.97 | 0.93 |
| DBP | *Ref.* | 0.70 | 0.65 | 0.76 |
| Current smoking | *Ref.* | 0.74 | 0.44 | 0.54 |
| Hypertension | *Ref.* | 0.50 | 0.59 | 1.00 |
| Diabetes | *Ref.* | 0.83 | 0.64 | 0.75 |
| Stroke | *Ref.* | 0.38 | 0.80 | 0.43 |
| LVEF | *Ref.* | 0.11 | 0.87 | 0.38 |
| SYNTAX Score | *Ref.* | 0.25 | 0.71 | 0.98 |
| rSS | *Ref.* | 0.27 | 0.091 | 0.42 |
| Stent | *Ref.* | 0.30 | 0.91 | 0.64 |
| P2Y12 inhibitor | *Ref.* | 1.00 | 0.66 | 0.88 |
| Statin | *Ref.* | 0.36 | 0.77 | 1.00 |
| ACEI/ARB/ARNI | *Ref.* | 1.00 | 1.00 | 0.86 |
| Beta blocker | *Ref.* | 0.74 | 1.00 | 1.00 |
| PCSK9i | *Ref.* | 1.00 | 0.70 | 1.00 |
| SGLT2i | *Ref.* | 1.00 | 0.40 | 0.85 |
| Hemoglobin | *Ref.* | 0.69 | 0.57 | 0.24 |
| Platelet | *Ref.* | 0.66 | 0.58 | 0.65 |
| LDL-C | *Ref.* | 0.58 | 0.37 | 0.90 |
| eGFR | *Ref.* | 0.67 | 0.64 | 0.86 |
| TNT | *Ref.* | 0.008 | 0.60 | 0.033 |

Abbreviations: SBP: systolic blood pressure, DBP: diastolic blood pressure, SYNTAX: SYNTAX score, rSS: residual SYNTAX score, LVEF: left ventricular ejection fraction, LDL-C: low-density lipoprotein cholesterol, ACEI: angiotensin-converting enzyme inhibitor, ARB: angiotensin receptor blocker, ARNI: angiotensin receptor and neprilysin inhibitor, PCSK9i: PCSK9 inhibitor, SGLT2i: sodium-glucose cotransporter 2 inhibitor.

**Supplementary Table 5.** Baseline Characteristics of Matched Cohorts Using the Low-Level Group as the Reference

|  | P value | | | |
| --- | --- | --- | --- | --- |
|  | TyG-BMI< 186 and BNP< 300 | TyG-BMI < 186 and BNP≥300 | TyG-BMI ≥ 186 and BNP< 300 | TyG-BMI ≥ 186 and BNP≥300 |
| Age | *Ref.* | 0.83 | 0.83 | 0.49 |
| Sex | *Ref.* | 0.66 | 0.43 | 0.73 |
| Heart rate | *Ref.* | 0.63 | 0.66 | 0.47 |
| SBP | *Ref.* | 0.41 | 0.95 | 0.92 |
| DBP | *Ref.* | 0.38 | 0.90 | 0.82 |
| Current smoking | *Ref.* | 1.00 | 0.69 | 0.62 |
| Hypertension | *Ref.* | 0.77 | 0.93 | 1.00 |
| Diabetes | *Ref.* | 0.62 | 0.64 | 0.50 |
| Stroke | *Ref.* | 1.00 | 1.00 | 0.45 |
| LVEF | *Ref.* | 0.11 | 0.80 | 0.57 |
| SYNTAX Score | *Ref.* | 0.41 | 0.70 | 0.73 |
| rSS | *Ref.* | 0.34 | 0.12 | 0.28 |
| Stent | *Ref.* | 0.099 | 0.59 | 0.51 |
| P2Y12 inhibitor | *Ref.* | 0.67 | 0.87 | 0.87 |
| Statin | *Ref.* | 1.00 | 0.69 | 1.00 |
| ACEI/ARB/ARNI | *Ref.* | 0.51 | 0.86 | 0.38 |
| Beta blocker | *Ref.* | 0.40 | 0.81 | 0.41 |
| PCSK9i | *Ref.* | 1.00 | 1.00 | 0.70 |
| SGLT2i | *Ref.* | 0.71 | 0.74 | 1.00 |
| Hemoglobin | *Ref.* | 0.86 | 0.074 | 0.84 |
| Platelet | *Ref.* | 0.73 | 0.98 | 0.84 |
| LDL-C | *Ref.* | 0.95 | 0.56 | 0.65 |
| eGFR | *Ref.* | 0.14 | 0.44 | 0.39 |
| TNT | *Ref.* | 0.008 | 0.64 | 0.051 |

Abbreviations: SBP: systolic blood pressure, DBP: diastolic blood pressure, SYNTAX: SYNTAX score, rSS: residual SYNTAX score, LVEF: left ventricular ejection fraction, LDL-C: low-density lipoprotein cholesterol, ACEI: angiotensin-converting enzyme inhibitor, ARB: angiotensin receptor blocker, ARNI: angiotensin receptor and neprilysin inhibitor, PCSK9i: PCSK9 inhibitor, SGLT2i: sodium-glucose cotransporter 2 inhibitor.
