# Supplementary figures and images for: Combined Triglyceride–Glucose and Triglyceride–Glucose–Body Mass Index with B-Type Natriuretic Peptide for Enhanced Prediction of Major Adverse Cardiovascular Events in ST-Elevation Myocardial Infarction Patients: A Retrospective Cohort Study
Source: Rev Cardiovasc Med. 2026 Jan 21;27(1):44062. doi: 10.31083/RCM44062 (PMC12873706; doi:10.31083/RCM44062)

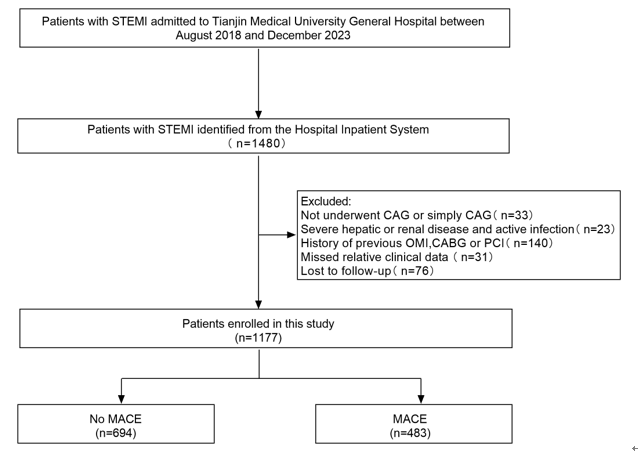

Supplement: Supplementary file 1 [file 2153-8174-27-1-44062-s1.zip › Supplementary fig1.png]

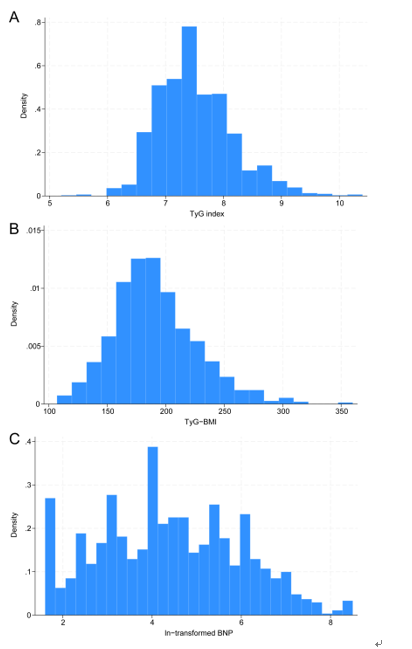

Supplement: Supplementary file 1 [file 2153-8174-27-1-44062-s1.zip › Supplementary fig2.png]
